# Left atrial enlargement is an independent predictor of stroke and systemic embolism in patients with non-valvular atrial fibrillation

**DOI:** 10.1038/srep31042

**Published:** 2016-08-03

**Authors:** Yasuhiro Hamatani, Hisashi Ogawa, Kensuke Takabayashi, Yugo Yamashita, Daisuke Takagi, Masahiro Esato, Yeong-Hwa Chun, Hikari Tsuji, Hiromichi Wada, Koji Hasegawa, Mitsuru Abe, Gregory Y. H. Lip, Masaharu Akao

**Affiliations:** 1 Department of Cardiology, National Hospital Organization Kyoto Medical Center, Kyoto, Japan; 2 Department of Arrhythmia, Ijinkai Takeda General Hospital, Kyoto, Japan; 3 Tsuji Clinic, Kyoto, Japan; 4 Division of Translational Research, National Hospital Organization Kyoto Medical Center, Kyoto, Japan; 5 Institute of Cardiovascular Sciences, University of Birmingham, United Kingdom; 6 Aalborg Thrombosis Research Unit, Department of Clinical Medicine, Aalborg University, Aalborg, Denmark

## Abstract

Controversy exists regarding whether left atrial enlargement (LAE) is a predictor of stroke/systemic embolism (SE) in atrial fibrillation (AF) patients. The Fushimi AF Registry, a community-based prospective survey, enrolled all AF patients in Fushmi-ku, Japan, from March 2011. Follow-up data and baseline echocardiographic data were available for 2,713 patients by August 2015. We compared backgrounds and incidence of events over a median follow-up of 976.5 days between patients with LAE (left atrial diameter > 45 mm; LAE group) and those without in the Fushimi AF Registry. The LAE group accounted for 39% (n = 1,049) of cohort. The LAE group was older and had longer AF duration, with more prevalent non-paroxysmal AF, higher CHADS_2_/CHA_2_DS_2_-VASc score, and oral anticoagulant (OAC) use. A higher risk of stroke/SE during follow-up in the LAE group was found (entire cohort; hazard ratio (HR): 1.92, 95% confidence interval (CI): 1.40–2.64; p < 0.01; without OAC; HR: 1.97, 95% CI: 1.18–3.25; p < 0.01; with OAC; HR: 1.83, 95% CI: 1.21–2.82; p < 0.01). LAE was independently associated with increased risk of stroke/SE (HR: 1.74, 95% CI: 1.25–2.42; p < 0.01) after adjustment by the components of CHA_2_DS_2_-VASc score and OAC use. In conclusion, LAE was an independent predictor of stroke/SE in large community cohort of AF patients.

Atrial fibrillation (AF) increases the risk of thromboembolism, such as stroke or systemic embolism (SE) fivefold^1^, but this risk is not homogeneous and depends on the various stroke risk factors^2^. These clinical risk factors are used to formulate clinical risk stratification schemes, such as CHADS_2_^3^ and CHA_2_DS_2_-VASc scores^4^. Echocardiographic parameters have been proposed as additional risk stratification refinements for predicting thromboembolism in AF patients. Left atrial structure and function may confer a risk for stroke/SE, given that thrombus formation mainly occurs in the left atrium^5^. In addition, left atrial structure and function represents AF burden or duration^6^, which might be related to increased risk of stroke/SE^7^.

Among many indices of left atrial structure and function, left atrial diameter determined by trans-thoracic echocardiography is the simplest method to measure, and used worldwide in daily clinical practice. Left atrial enlargement (LAE), defined as an enlarged left atrial diameter, has been reported to be a predictor of stroke/SE in AF patients in some^8^,^9^,^10^,^11^,^12^, but not all studies^13^,^14^,^15^,^16^, and data from large-scale prospective cohorts are limited. The aim of this study is to investigate the impact of LAE on the incidence of stroke/SE in a large community-based prospective survey of Japanese AF patients, the Fushimi AF Registry.

## Methods

The Fushimi AF Registry is a community-based prospective survey of AF patients in Fushimi-ku, Japan^17^,^18^. The detailed study design, patient enrollment, the definition of the measurements, and subjects’ baseline clinical characteristics of the Fushimi AF Registry were previously described^17^. The inclusion criterion for the registry is the documentation of AF on a 12-lead electrocardiogram or Holter monitoring at any time. There are no exclusion criteria. A total of 80 institutions participated in the registry. We started to enroll patients from March 2011. All of the participating institutions attempted to enroll all consecutive patients with AF under regular outpatient care or under admission.

Among the registry participants, we analyzed the patients whose follow-up data were available. The primary endpoint in this analysis was the incidence of stroke/SE during follow-up period. Other clinical endpoints included the incidence of ischemic stroke, hemorrhagic stroke, all-cause death, cardiac death, non-cardiac death, and composite endpoints of ‘stroke, SE, and all-cause death’ during follow-up period. The type of AF was classified into 2 groups; paroxysmal AF and sustained AF which was defined as the combination of persistent AF and permanent AF^19^. AF duration was calculated from the day of estimated AF documentation to the day of entry into the registry.

Echocardiographic data were collected at the time of entry into the registry. Left atrial diameter was measured using M-mode or two-dimensional echocardiography, from the posterior aortic wall to the posterior left atrial wall, in the parasternal long axis view at the end-ventricular systole^20^. We simply defined LAE as left atrial diameter more than 45 mm, according to the previous study^16^. We did not categorize left atrial diameter as gender-specific variable, since mean left atrial diameter was not statistically different between male and female in our registry.

We compared the background and incidence of clinical events during follow-up period between those with left atrial diameter > 45 mm (LAE group) and those without it (non-LAE group). We excluded registry participants for whom data on their left atrial diameter were missing, those with mitral stenosis and/or prior cardiac valve surgery.

### Statistical analysis

Continuous variables are expressed as mean ± standard deviation, or median and interquartile range according to the distributions. Categorical variables are presented as numbers and percentages. We compared categorical variables using the chi-square test when appropriate; otherwise, we used Fisher’s exact test. We compared continuous variables using Student’s t-test or Wilcoxon rank-sum test on the basis of the distribution. The Kaplan-Meier method was used to estimate the cumulative incidences of clinical events. Data were analyzed as crude and stratified by oral anticoagulant (OAC) prescription at baseline. Data were also stratified by major subgroups which were considered to be confounders. We carried out multivariate analysis using a Cox proportional hazards model. The covariates chosen to be included were LAE, OAC prescription at baseline, and components of the CHA_2_DS_2_-VASc risk score^4^. We used JMP version 10 (SAS Institute, Cary, NC) to perform all of these analyses. Two-sided P values less than 0.05 were considered statistically significant.

### Ethics

The study protocol conforms to the ethical guidelines of the 1975 Declaration of Helsinki, and was approved by the ethical committees of the National Hospital Organization Kyoto Medical Center and Ijinkai Takeda General Hospital. Since the present research involves an observational study not using human biological specimens, written informed consent was not obtained from each patient for their clinical records to be used in this study, according to the ethical guidelines for epidemiological research issued by the Ministry of Education, Culture, Sports, Science and Technology and the Ministry of Health, Labour and Welfare, Japan.

## Results

A total of 4,392 patients were enrolled in the Fushimi AF Registry by August 2015. Of 4,124 patients who were enrolled one year before, follow-up data (collected every year) were available for 3,713 patients (follow-up rate: 90.0%). Of these 3,713 patients, we excluded 191 patients with mitral stenosis or prior cardiac valve surgery. Among 3,522 non-valvular AF patients, left atrial echocardiographic data were available for 2,713 patients, and left atrial diameter data were missing for 809 patients. Age and gender were comparable between patients with and without left atrial diameter data (mean age 73.7 vs 73.7 years; p = 0.99, female sex 40% vs. 41%, p = 0.57, respectively). Patients without left atrial diameter data had lower CHADS_2_ score (1.93 vs. 2.05; p = 0.03), CHA_2_DS_2_-VASc score (3.24 vs. 3.39; p = 0.03), and had lower prescription of OAC at baseline (45% vs. 55%; p < 0.01). Cumulative incidence of stroke/SE during follow-up period was comparable between the two groups (5.6% vs. 5.7%; p = 0.90).

**Figure 1:**
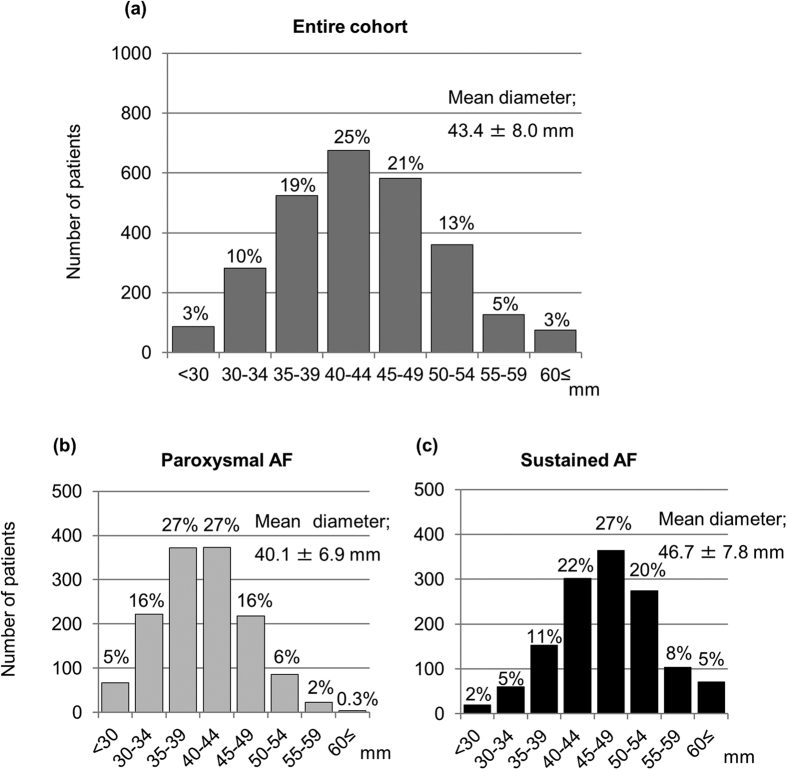
Distributions of left atrial diameter. (**a**) Entire cohort. (**b**) Patients with paroxysmal atrial fibrillation (AF). (**c**) Patients with sustained AF. AF = atrial fibrillation.

A total of 2,713 patients were included in the analysis. Median follow-up period was 976.5 days (interquartile range: 468 to 1190 days). The distributions of left atrial diameter are shown in Fig. 1. Patients in the LAE group accounted for 39% (n = 1,049) of all patients. Patients with sustained AF had larger left atrial diameter than those with paroxysmal AF (46.7 ± 7.8 vs. 40.1 ± 6.9 mm; p < 0.01).

### LAE vs non-LAE patients

Baseline characteristics are shown in Table 1. The LAE group was older, had higher CHADS_2_ and CHA_2_DS_2_-VASc scores (all p < 0.01). Left atrial diameter was correlated with AF duration (p < 0.01). The LAE group had higher use of OAC at baseline (p < 0.01), and OAC prescription data at baseline were missing for 13 patients. Patients without OAC were more often female (p < 0.01), younger (p = 0.03), had lower CHADS_2_ score and CHA_2_DS_2_-VASc score (both p < 0.01), and had smaller left atrial diameter (p < 0.01).

### Outcomes

Major clinical cumulative events in the entire cohort during follow-up period were as follows: stroke/SE in 154, ischemic stroke in 112, hemorrhagic stroke in 43, SE in 4, all-cause death in 418, cardiac death in 64, non-cardiac death in 354, and stroke/SE/all-cause death in 530, respectively. Major clinical events in the entire cohort, patients without OAC, and those with OAC are shown in Table 2.

**Figure 2:**
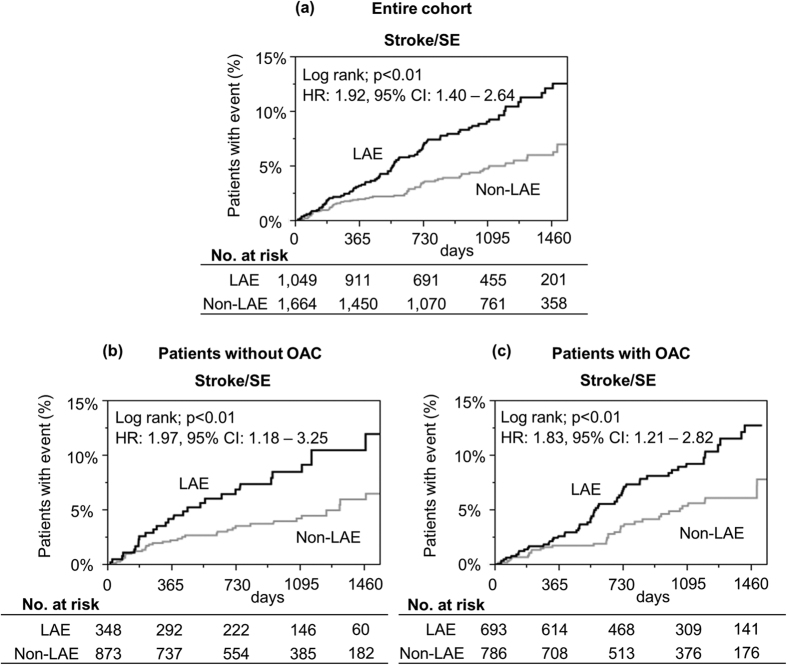
Kaplan-Meier curves for the incidence of stroke or systemic embolism (SE) during follow-up period. (**a**) Entire cohort, (**b**) Patients without oral anticoagulant (OAC). (**c**) Patients with OAC at baseline. CI = confidence interval, HR = hazard ratio, LAE = left atrial enlargement, OAC = oral anticoagulant, SE = systemic embolism.

**Figure 3:**
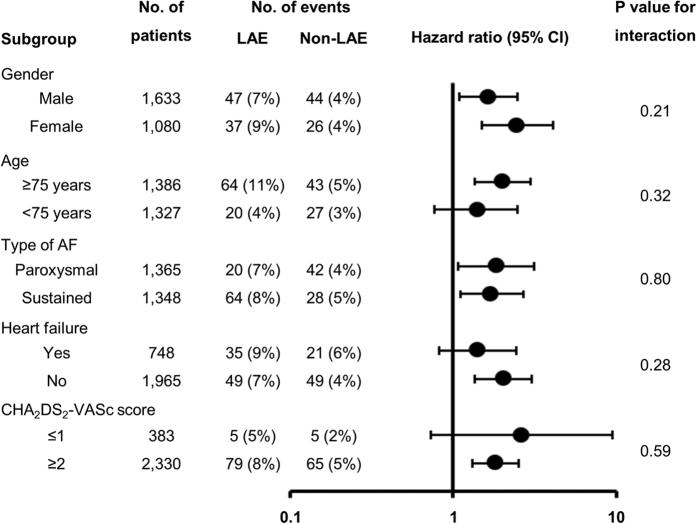
Impact of left atrial enlargement (left atrial diameter > 45 mm) on the incidence of stroke or systemic embolism, according to major subgroups. AF = atrial fibrillation, CI = confidence interval, LAE = left atrial enlargement.

Kaplan-Meier curves for the incidence of stroke/SE are shown in Fig. 2. A higher risk of stroke/SE during follow-up period in the LAE group was consistently observed in the entire cohort, and in patients stratified by OAC prescription at baseline (entire cohort; hazard ratio (HR): 1.92, 95% confidence interval (CI): 1.40–2.64; p < 0.01, patients without OAC; HR: 1.97, 95% CI: 1.18–3.25; p < 0.01, and patients with OAC; HR: 1.83, 95% CI: 1.21–2.82; p < 0.01, respectively). Statistical tests for interaction were not significant for all of the major subgroups (Fig. 3).

### Multivariate analysis/Sensitivity analyses

**Figure 4:**
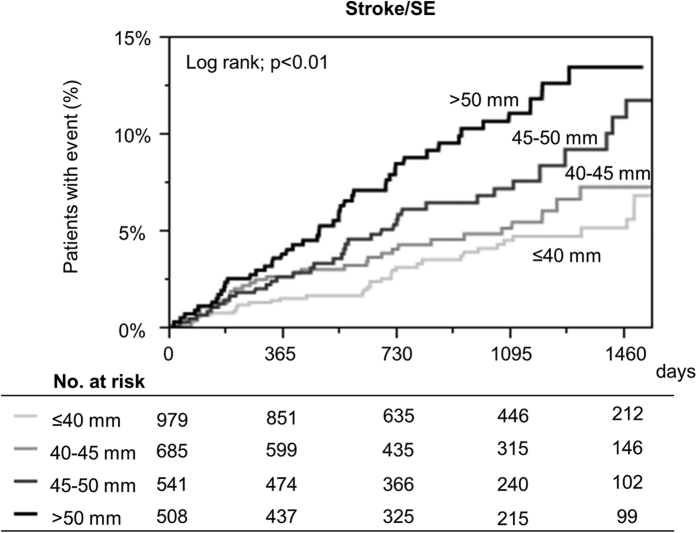
Kaplan-Meier curves for the incidence of stroke or systemic embolism (SE) according to 4 left atrial diameter strata (≤40 mm vs. 40–45 mm vs. 45–50 mm vs. >50 mm). SE = systemic embolism.

On multivariate analysis, after adjustment by the components of CHA_2_DS_2_-VASc score and OAC prescription at baseline, LAE emerged as an independent risk factor of the incidence of stroke/SE (HR: 1.74, 95% CI: 1.25–2.42; p < 0.01) (Table 3). LAE still remained a significant risk factor of stroke/SE, when age was included as continuous variable on multivariate analysis (HR: 1.73, 95% CI: 1.24–2.41; p < 0.01). When we defined the cut-off value of left atrial diameter as more than 50 mm, left atrial diameter > 50 mm was also independently associated with higher risk of stroke/SE on multivariate analysis (HR: 1.69, 95% CI: 1.18–2.41; p < 0.01). Kaplan-Meier curves for the incidence of stroke/SE between ordinal variable of 4 left atrial diameter strata (≤ 40 mm, 40–45 mm, 45–50 mm, > 50 mm) are shown in Fig. 4. Increasing left atrial diameter could stratify the incidence of stroke/SE during follow-up period (log-rank; p < 0.01). When we used left atrial diameter indexed by body surface area, indexed left atrial diameter still remained significantly associated with a higher risk of stroke/SE (HR: 1.08 per 1 mm/m^2^, 95% CI: 1.05–1.10; p < 0.01).

We also performed multivariate analysis among patients without OAC at baseline. LAE remained an independent risk factor of stroke/SE (HR: 1.85, 95% CI: 1.10–3.10; p = 0.02). Left atrial diameter > 50 mm was also independently associated with higher risk of stroke/SE (HR: 2.22, 95% CI: 1.19–3.96; p = 0.01), and ordinal variable of 4 left atrial diameter strata (≤40 mm, 40–45 mm, 45–50 mm, >50 mm) could also stratify the incidence of stroke/SE during follow-up (log-rank; p < 0.01).

## Discussion

In the contemporary large community-based prospective study, we have shown that LAE was an independent predictor of stroke/SE in non-valvular AF patients. This relationship remained, even after adjustment for differences in baseline characteristics and was consistent whether on OAC or not taking OAC.

A relationship between LAE and increased risk of stroke/SE in AF patients was first proposed in the 1980s^8^,^9^. In early 1990s, some reports supported that LAE was significantly associated with increased risk of stroke/SE^10^,^11^,^12^, while other studies found that LAE could not predict stroke events^13^,^14^. However, these studies were small-sized and retrospective in design.

In late 1990s and 2000s, sub-analyses of clinical trials reported that left atrial diameter was not independently associated with thromboembolic events^15^,^16^. In the sub-analysis of non-anticoagulated AF patients from 3 clinical trials, a total of 1,066 patients were included, and ischemic stroke occurred in 78 patients during a mean period of 1.6 years. In this analysis, left atrial diameter tended to be associated with stroke (relative risk, 1.02 per 1mm; p = 0.10); however, it did not reach a statistical significance^15^. In a sub-analysis of AFFIRM trial^21^, a total of 2,334 patients with left atrial diameter data were included, and stroke occurred in 86 patients during a mean period of 1,298 days. This study concluded that left atrial diameter was not a predictor of stroke; however, most of the patients included in the AFFIRM trial were prescribed OAC, and patients with excessively enlarged left atrium might have been excluded (31% of patients had left atrial diameter greater than 45 mm, compared to 39% in our study)^16^.

More recent studies have reported that LAE was independently associated with increased risk of stroke after adjustment by the CHA_2_DS_2-_VASc score, although endpoints in these studies were the transesophageal echocardiographic surrogate markers of stroke^22^,^23^. Thus, controversy exists regarding whether LAE is a significant predictor for the incidence of stroke/SE independent from established risk factors, and there have been no data with large-scale, prospective studies with long term follow-up. To the best of our knowledge, our study is one of the largest ‘all-comers” studies, providing longer-term follow-up data about the association between LAE and the incidence of stroke/SE in AF patients, and providing robust evidence that LAE was independently associated with increased risk of stroke/SE.

Several mechanisms could explain how LAE is associated with higher risk of stroke/SE in AF patients. First, LAE promotes blood stasis, which in turn predisposes to thrombus formation and the potential for the embolization. Indeed, transesophageal echocardiography studies have found that LAE was associated with spontaneous echo contrast in left atrium^22^. Second, left atrial diameter can be related to AF duration^24^, or arrhythmia burden^6^. Cardiac implanted electronic device-detected AF burden was reportedly associated with increased risk of stroke/SE^7^. However, AF burden is difficult to measure in patients without cardiac implanted electronic device. Indeed, left atrial diameter could be a good marker of AF burden/duration, and assessment of left atrial size may add important incremental information in the evaluation of stroke/SE risk in AF patients.

### Study Limitations

First, this is an observational study and provides only associative evidence, not causative. We cannot rule out the possibility of unmeasured confounders. Second, we measured only left atrial anterior-posterior diameter. Left atrial volume is a more reliable estimator of left atrial size, and has been recommended when assessing left atrial size, since left atrial dilatation can be eccentric^20^. However, left atrial diameter is more readily available and more widely employed in daily clinical practice^25^. Third, among 3,522 patients, left atrial echocardiographic data were missing in 809 patients, leading to selection bias. However, patients without left atrial diameter data were not particularly skewed in our study, considering that the backgrounds and incidence of stroke/SE were comparable between patients with and without echocardiographic data. Fourth, AF duration data were available for only 1,771 patients among the entire cohort, and were mostly based on the physician’s judgement or patient’s self-report. Fifth, antithrombotic therapy were selected at the discretion of the attending physician, and we stratified the entire cohort by OAC prescription only at baseline. Finally, we did not measure 24-hour blood pressure and collected blood pressure data only at the time of entry into the registry, notwithstanding hypertension being associated with the incidence of AF^26^, and the incidence of thromboembolism among AF patients^27^.

## Conclusions

In this large community cohort of Japanese patients with AF, LAE was an independent predictor of stroke/SE, suggesting this simple echocardiographic parameter could refine thromboembolic risk stratification of AF patients.

## Additional Information

**How to cite this article**: Hamatani, Y. *et al*. Left atrial enlargement is an independent predictor of stroke and systemic embolism in patients with non-valvular atrial fibrillation. *Sci. Rep.* **6**, 31042; doi: 10.1038/srep31042 (2016).

## Figures and Tables

**Table 1:** Baseline characteristics and Co-morbidities.

Number	LAE	Non-LAE	p value	Without OAC	With OAC	p value
	1,049	1,664		1,221	1,479	
**Baseline characteristics**						
Female	411 (39%)	669 (40%)	0.60	526 (43%)	550 (37%)	<0.01
Age (years)	74.9 ± 10.2	72.9 ± 11.2	<0.01	73.2 ± 12.5	74.1 ± 9.2	0.03
Age ≥ 75 years	591 (56%)	795 (48%)	<0.01	589 (48%)	786 (53%)	0.01
Body mass index (kg/m^2^)	24.0 ± 4.2	22.4 ± 3.8	<0.01	22.7 ± 4.0	23.3 ± 4.0	<0.01
Body weight (kg)	61.8 ± 14.4	57.7 ± 12.5	<0.01	58.1 ± 13.5	60.3 ± 13.3	<0.01
Systolic blood pressure (mmHg)	123.9 ± 20.3	124.1 ± 18.9	0.85	125.1 ± 19.4	123.0 ± 19.4	<0.01
Diastolic blood pressure (mmHg)	69.9 ± 13.6	70.1 ± 12.5	0.74	70.1 ± 13.0	69.9 ± 12.9	0.57
Heart rate (beats/min)	77.9 ± 15.6	77.9 ± 15.8	0.96	78.6 ± 16.2	77.4 ± 15.3	0.04
Paroxysmal AF	288 (27%)	1,077 (65%)	<0.01	769 (63%)	591 (40%)	<0.01
AF duration (months)	43.5 (10.0, 91.3)	21.0 (4.0, 59.0)	<0.01	25.0 (4.0, 62.0)	31.0 (7.0, 75.0)	<0.01
Smoking history	354 (34%)	597 (36%)	0.26	413 (48%)	535 (51%)	0.20
Previous catheter ablation	46 (4%)	136 (8%)	<0.01	86 (7%)	95 (6%)	0.52
Calculated CrCl (ml/min)	56.6 (35.9, 76.5)	58.6 (39.7, 80.2)	0.02	59.6 (36.0, 82.1)	56.6 (39.9, 76.2)	0.11
Hemoglobin (g/dL)	12.9 ± 2.1	12.9 ± 2.0	0.65	12.7 ± 2.1	13.0 ± 2.0	<0.01
**Co-morbidities**						
CHADS_2_ score	2.26 ± 1.30	1.92 ± 1.36	<0.01	1.77 ± 1.34	2.27 ± 1.31	<0.01
CHA_2_DS_2_-VASc score	3.64 ± 1.65	3.24 ± 1.74	<0.01	3.11 ± 1.77	3.63 ± 1.63	<0.01
History of stroke/SE	225 (21%)	348 (21%)	0.74	175 (14%)	396 (27%)	<0.01
Heart failure	390 (37%)	358 (22%)	<0.01	259 (21%)	484 (33%)	<0.01
Hypertension	691 (66%)	997 (60%)	<0.01	722 (59%)	957 (65%)	<0.01
Diabetes mellitus	263 (25%)	376 (23%)	0.14	261 (21%)	374 (25%)	0.02
Dyslipidemia	444 (42%)	713 (43%)	0.79	500 (41%)	653 (44%)	0.09
Vascular disease	117 (11%)	165 (10%)	0.30	130 (11%)	152 (10%)	0.75
Chronic kidney disease	445 (42%)	564 (34%)	<0.01	411 (34%)	593 (40%)	<0.01
COPD	56 (5%)	87 (5%)	0.90	57 (5%)	84 (6%)	0.24
History of major bleeding	43 (4%)	71 (4%)	0.83	58 (5%)	55 (4%)	0.18
**OAC prescription at baseline**	693 (66%)	786 (47%)	<0.01	0 (0%)	1,479 (100%)	<0.01
Warfarin	611 (59%)	655 (39%)	<0.01	0 (0%)	1,266 (86%)	<0.01
Non-vitamin K antagonist OAC	82 (8%)	131 (8%)	0.96	0 (0%)	213 (14%)	<0.01
**Echocardiography**						
LV ejection fraction (%)	61.4 ± 12.2	64.2 ± 11.1	<0.01	63.9 ± 11.1	62.5 ± 12.0	<0.01
LV ejection fraction ≤ 40%	70 (7%)	82 (5%)	0.05	60 (5%)	92 (6%)	0.15
LV end-diastolic dimension (mm)	48.2 ± 6.9	45.5 ± 5.7	<0.01	45.9 ± 6.2	47.0 ± 6.4	<0.01
LV end-systolic dimension (mm)	32.2 ± 7.5	29.4 ± 5.9	<0.01	29.8 ± 6.3	31.0 ± 7.0	<0.01
LV septal wall thickness (mm)	9.8 ± 1.9	9.3 ± 1.6	<0.01	9.4 ± 1.6	9.6 ± 1.8	<0.01
LV posterior wall thickness (mm)	9.8 ± 1.6	9.2 ± 1.4	<0.01	9.3 ± 1.4	9.5 ± 1.5	<0.01
Left atrial diameter (mm)	51.3 ± 5.4	38.4 ± 4.8	<0.01	41.5 ± 7.8	44.9 ± 7.9	<0.01

Categorical data are presented as number (%). Continuous data are presented as mean ± standard deviation (SD), or median and interquartile range (25%, 75%) according to the distribution. AF = atrial fibrillation, COPD = chronic obstructive pulmonary disease, CrCl = creatinine clearance, LAE = left atrial enlargement, LV = left ventricular, OAC = oral anticoagulant, SE: systemic embolism.

**Table 2:** Incidence of clinical events during follow-up (/100 person-years).

Outcomes	Entire cohort		Patients without OAC		Patients with OAC	
	Incidence rate	p value*	Incidence rate	p value*	Incidence rate	p value*
Stroke/SE	2.4		2.2		2.5	
LAE	3.4	<0.01	3.4	<0.01	3.3	<0.01
Non-LAE	1.7		1.7		1.8	
Ischemic stroke	1.7		1.4		1.9	
LAE	2.4	<0.01	2.2	0.02	2.6	0.01
Non-LAE	1.2		1.1		1.3	
Hemorrhagic stroke	0.6		0.7		0.6	
LAE	0.9	0.046	1.0	0.26	0.8	0.11
Non-LAE	0.5		0.6		0.4	
All-cause death	6.3		7.5		5.3	
LAE	7.0	0.05	8.3	0.31	6.3	<0.01
Non-LAE	5.8		7.2		4.4	
Cardiac death	1.0		0.9		1.0	
LAE	1.1	0.40	1.1	0.48	1.1	0.67
Non-LAE	0.9		0.8		0.9	
Non-cardiac death	5.3		6.6		4.3	
LAE	5.9	0.08	7.2	0.41	5.2	<0.01
Non-LAE	4.9		6.3		3.4	
Stroke/SE/All-cause death	8.1		9.3		7.2	
LAE	9.5	<0.01	11.0	0.07	8.6	<0.01
Non-LAE	7.3		8.7		5.3	

*p value for comparison between LAE versus non-LAE from log-rank test. LAE = left atrial enlargement, OAC = oral anticoagulant, SE = systemic embolism.

**Table 3:** Indicators of the incidence of stroke or systemic embolism during follow-up. Multivariate analysis.

Variable	HR (95% CI)	p value
**Left atrial enlargement (>45** **mm)**	**1.74 (1.25–2.42)**	**<0.01**
OAC prescription	0.88 (0.63**–**1.24)	0.46
Heart failure	1.30 (0.91**–**1.83)	0.15
Hypertension	0.98 (0.70**–**1.37)	0.88
Age ≥ 75 years (vs. <65 years)	2.26 (1.35**–**4.03)	<0.01
Age 65–74 years (vs. <65 years)	0.99 (0.55**–**1.86)	0.97
Diabetes mellitus	1.01 (0.70**–**1.45)	0.94
History of stroke/SE	1.55 (1.08**–**2.20)	0.02
Vascular disease	1.10 (0.66**–**1.73)	0.71
Female sex	0.93 (0.66**–**1.30)	0.68

AF = atrial fibrillation, CI = confidence interval, HR = hazard ratio, OAC = oral anticoagulant, SE = systemic embolism.
